# Public support for gun restrictions in public places that serve alcohol: the cost of enforcement matters

**DOI:** 10.1093/haschl/qxag200

**Published:** 2026-08-10

**Authors:** Robert Zeithammer, Supriya Kapur, Diana Silver, James Macinko

**Affiliations:** UCLA Anderson School of Management, Los Angeles, CA 90095, United States; NYU Department of Public Health Policy and Management, School of Global Public Health, New York, NY 10003, United States; NYU Department of Public Health Policy and Management, School of Global Public Health, New York, NY 10003, United States; UCLA Department of Health Policy and Management, Fielding School of Public Health, Los Angeles, CA 90095, United States

**Keywords:** gun policy, alcohol, political support, opinion polls

## Abstract

**Introduction:**

Public opinion on firearm legislation varies, though there is broader agreement on restricting guns in alcohol-serving venues.

**Methods:**

This study used a discrete choice experiment with 306 US adults in June 2025 to examine preferences for firearm restrictions across different conditions. Scenarios varied by who would be restricted (felons, currently intoxicated individuals, or those with prior alcohol-related offenses), venue size (small, medium, or large), and annual enforcement costs ranging from $0 to $300.

**Results:**

Results show that when no enforcement costs are included, a majority of the respondent sample weighted to voting-eligible Americans (56%-74%, depending on the restricted group and venue size) supports firearm restrictions, with support exceeding 65% for restrictions in large venues. However, introducing a $100 annual cost reduces support: significant majority support remains only for restricting combinations of felons, intoxicated people, or those with alcohol-related convictions and only in large venues. Support patterns differ by demographics: women show the strongest support regardless of political affiliation, Democratic men show moderate support, while Republican men and Independents are least supportive. Gun ownership did not independently affect preferences.

**Conclusions:**

While support for firearm restrictions in alcohol-serving venues is widespread, even modest implementation costs significantly reduce public backing.

Key points

- While a majority of respondents favor firearm restrictions in alcohol-serving venues, support is stronger for larger venues and varies modestly by the targeted population.
- Introducing even modest individual costs (eg, $100 annually) substantially reduces majority support, highlighting the importance of cost framing in legislative proposals.
- Support is more strongly associated with gender and political affiliation (highest among women across political parties) than firearm ownership status.

## Introduction

Firearms are a major source of preventable morbidity and mortality in the United States, contributing to over 44 000 deaths in 2024 and generating societal costs estimated to be in excess of $500 billion.^1,2^ There is considerable evidence that alcohol contributes to firearm-related harms, including homicide, suicide, intimate partner violence, and unintentional deaths.^3^ A systematic review found that among those recently deceased due to firearms, up to 25% had consumed alcohol prior to their deaths, and there was a positive relationship between alcohol sales and firearm assault.^3^

To mitigate the increased firearm risk due to alcohol, some states regulate the intersection of alcohol and firearms by explicitly targeting high-risk individuals (such as those with a previous DUI conviction), allowing alcohol abuse to be a condition for issuance of an Extreme Risk Protective Order (ERPO), or banning firearms from locations (such as bars or sports stadiums) where firearms and alcohol are more likely to mix.^4^

A recently published analysis of the current legal landscape found 10 different types of alcohol-related firearm laws, with nearly every state having passed at least 1 of these types of laws.^5^ The legal analysis also showed considerable state-to-state variation in how such laws were formulated, how the targets of action (individuals, locations) were defined, and penalties for noncompliance.^5^ For example, Texas prohibits firearms only from establishments deriving 51% or more of revenue from alcohol sales, while neighboring New Mexico bans concealed carry in any premises licensed to sell alcohol for on-site consumption.^5^

This paper analyzes the public support for different variations of restrictions that ban certain types of individuals from firearms from public places that serve alcohol. While general gun restrictions have become increasingly polarized by political affiliation and gender,^6,7^ opinion polls generally find strong public support for specifically regulating the possession of firearms where alcohol is consumed. For example, a nationally representative survey from 2023 found that 77% of non-gun owners and 70% of gun owners supported prohibiting the carrying of guns in bars.^8^ While opinion polls suggest the regulations this paper studies are broadly popular, simple polling rarely includes the costs of implementing and enforcing such laws and obscures tradeoffs that may concern respondents.

In order to more closely model the type of choice voters are confronted with that explicitly brings in the cost of enforcement, we developed and fielded a Discrete Choice Experiment (DCE) that gives respondents a choice between ballot measures that differ both in the type of bans they enact and in the added taxpayer costs the enforcement of such bans would involve. The DCE allows us to assess both overall support for different restrictions, as well as the differences in such support across demographic and psychographic individual characteristics.

## Methods

We conducted a DCE where participants were presented with a set of ballot-measure scenarios to choose from and differed based on a set of experimentally manipulated firearm restrictions each measure would entail, and the additional tax the measure's enforcement would cost each taxpayer.^9,10^ Discrete choice experiments are a stated preference method based on economic utility theory and are a robust approach to systematically assess tradeoffs and priorities among an individual's preferences for different aspects of products or services.^10^ Discrete choice experiment results model people's utilities for alternatives in terms of “partworth” utilities for the multiple features (“attributes”) that together describe a product or service.^9^ Discrete choice experiments are commonly applied to the design of medical treatments,^11,12^ public policies,^13,14^ and job types, among other areas.^15^ The method is grounded in microeconomic utility-maximization theory, and results can accurately predict subsequent behaviors in complex arenas including health-care choices.^16-20^ Different from opinion polls, DCEs require that participants make explicit choices among different scenarios to capture tradeoffs across different dimensions of the policy in question.^21^

Respondents were presented with a hypothetical scenario regarding 2 potential ballot measures. Each individual was asked:

*Please imagine the 2026 midterm election has the following 2 measures “A” and “B” on the ballot. These measures differ in terms of which type of person is prohibited from possessing a firearm in which type of public place, and the additional tax associated with its enforcement*.

*If these were your only options, which ballot measure would you choose?*

The DCE involved 4 attributes: restrictions in 3 different venue sizes and the cost of enforcement. For each type of place where alcohol is served (small venues like bars, medium venues like movie theaters, and large venues like sports stadiums), the first 3 attributes specified who—if anyone—is restricted from possessing a firearm in that place. There were 6 options: no restrictions, visibly intoxicated persons, those with a previous alcohol-related conviction, those with a previous felony, either intoxicated or felon, or either intoxicated or alcohol-related conviction. These venue types and restricted groups mirror the main dimensions along which existing state alcohol-related firearm laws vary, such as which locations are covered and which persons are targeted (eg, possession while intoxicated, alcohol-related conviction histories, and prohibited-person categories), as documented in the dataset described above.^5^ The fourth attribute was the individual's share of the annual cost of enforcing the new law ($0, $100, $200, or $300 per year) framed as a tax increase. Because per-person costs of enforcing venue-based firearm restrictions are not well documented, these levels were chosen to include a $0 reference level directly comparable to conventional opinion polls, to use simple round increments, and to span a policy-relevant range of hypothetical costs well below recent estimates of Americans’ mean willingness to pay (WTP) for a 20% national reduction in gun violence ($744 per year).^22^

At the beginning of the survey, each of these terms was defined, and respondents had to answer comprehension checks to ensure they understood the definition of each term. Once qualified, each respondent completed 12 choice tasks, presented in random order, followed by demographic and other questions. Each task presented 2 experimentally constructed ballot measures (“A” and “B”) plus a fixed opt-out alternative. Each measure was described by the 4 attributes described in the previous paragraph. Because the full factorial (6 × 6 × 6 × 4 = 864 profiles) exceeds what any 1 respondent could evaluate, profiles were generated with Sawtooth Software's fractional design, with a different randomized design version assigned to each respondent. For each scenario, participants were also given the choice of “None of the above. I would not support either of these measures.” See Appendix Figure S1 for an example. The “None of the above” option is not only realistic but also establishes a clear uniform benchmark against which the preferences for the different restrictions are measured. Because “no restrictions” could also appear as a level within a ballot measure for all venues, a measure could impose a tax while restricting no group from possessing firearms in any venue. Support for such measures isolates respondents’ baseline aversion to the tax itself.

### Study sample

Adult (aged 18 and above) participants were recruited from CloudResearch's Connect online research platform.^23,24^ During June 2025, 537 people entered the survey. After eliminating those who did not provide consent, those who failed comprehension checks, and the fastest 5%, the responses of 306 individuals were analyzed.

In addition to choices in the main DCE tasks, demographic characteristics (age, birth, sex), educational attainment (high school or less vs some college or more), political party identification (Republican, Independent/other, Democrat), gun ownership and attitudes (whether the individual had a firearm in their household and whether the individual worried about becoming a victim of firearm violence), and state of residence were collected from each respondent. Based on state of residence, we then linked the 2024 Giffords Annual Gun Law Scorecard and the state's level of population density obtained from the Census Bureau to each individual's responses.

To estimate representative population- and subgroup-level summaries, we employ a weighting scheme designed to match known voting results in the 2024 Presidential election where Trump and Harris were 49.8% and 48.3% with a 63.7% turnout, so the weights are 1.11 for Trump Voters, 0.63 for Harris Voters, 0.41 for third-party voters, and 1.82 for nonvoters. We prioritize weighting on vote choice and turnout because the outcome of interest is a hypothetical ballot measure and gun policy attitudes are strongly polarized along political lines. Because our target population is the voting-eligible adult population, nonvoters were retained and up-weighted rather than excluded. A standard way to summarize the effect of a weighting scheme on the statistical precision of the resulting weighted averages is to compute the effective sample size—the equivalent number of unweighted samples that would provide the same level of precision as a weighted sample.^25^ The effective sample size of qualified respondents is 252.

As a sensitivity test, we re-estimated all population-level summaries using alternative weights raked to census benchmarks for age group and sex (Appendix Figures A1 and A2). Our data under-samples older people, so the alternative weighting scheme results in skewed weights and a reduced effective sample size. Nevertheless, our main findings remain robust to the choice of weighting scheme. Results presented in the main text use the recent voting weighting scheme.

Participants indicated their free and informed consent to participate in the study and received a modest payment. The study protocol was reviewed by the UCLA Institutional Review Board and deemed exempt from full review.

### Statistical analysis

A Mixed Logit Model with a Multivariate Normal population distribution of individual-level utility parameters was used to model the choice among the alternatives in each task in 2025-2026.^26^ The model was estimated using what Sawtooth Software refers to as a Hierarchical-Bayes Multinomial Logit Model.^27,28^ The output of the estimation is individual-level “partworth” utilities. Based on these measures, we computed importance weights and deterrence probabilities to facilitate interpretation. See the statistical Appendix for a detailed explanation of these quantities. The hierarchical structure accounts for repeated choices by the same respondent. Each respondent's 12 choices inform their own utility, which is partially pooled toward the population distribution. The opt-out was modeled as an additional alternative with its own utility.

## Results

Table A1 describes the sample taken for analysis and compares its characteristics to the US population. The sample is representative of the US population in terms of gun ownership, census region, and attitudes toward guns. However, we undersample older people, Republicans, Trump voters, and people without a high school degree.

Table 1 shows average importance weights, both overall and by respondent subgroup. Focusing first on overall importance, we find a small increase in importance for regulating larger venues: differences in who can bring a firearm to a bar imply an importance weight of 21.6%, while the same differences have an importance of 22.9% in theaters and 26.7% in large venues. There is no difference in average importance of regulating different venue sizes between gun owners and non-owners. See Appendix Table S2 for more details.

**Table 1.** Importance weights by venue type and respondent characteristics.

Respondent group	Bars	Theaters	Large venues	Tax
All respondents	21.6	22.9	26.7	28.8
(SE)	0.6	0.6	0.7	0.6
Democrats	21.6	23.7	28.1	26.6
(SE)	0.8	0.8	0.8	0.8
Republicans	21.2	21.4	24.5	32.9
(SE)	1.0	1.0	1.1	1.3
Independents	21.9	23.0	26.5	28.6
(SE)	0.9	1.0	1.0	1.1
Women	22.0	22.5	26.4	29.1
(SE)	0.7	0.8	0.9	0.9
Men	21.3	23.1	26.8	28.8
(SE)	0.8	0.8	0.8	0.8
No gun in household	21.6	23.2	27.0	28.2
(SE)	0.7	0.7	0.8	0.7
≥1 gun in household	21.6	22.1	26.1	30.2
(SE)	0.8	0.8	1.0	1.0

Cell entries are group means (and standard errors). Results weighted to reflect the 2024 Presidential vote share.

**Figure 1.**
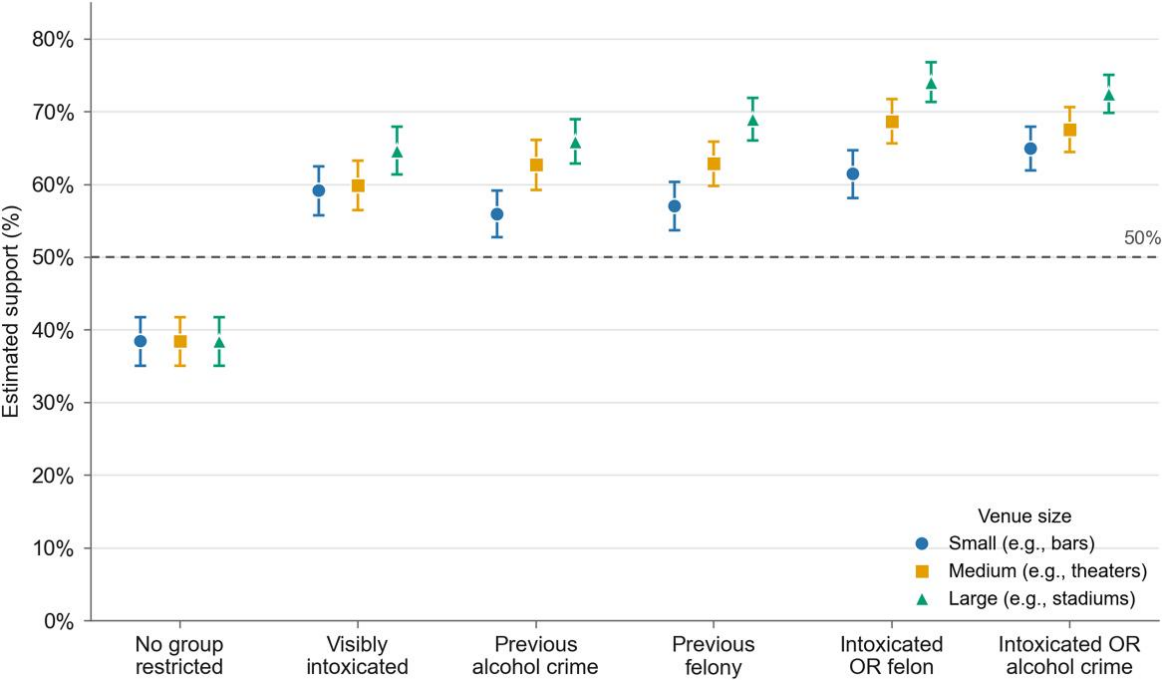
Weighted estimates of the percent of US adults supporting firearm restrictions when they are free to enforce, by restricted group and venue size. Notes: Points are weighted mean predicted support for a single-venue measure (with no restrictions in the other 2 venue types), whiskers are standard errors. The “no group restricted” estimate is identical across venue sizes by construction. Dashed line marks 50% support.

**Figure 2.**
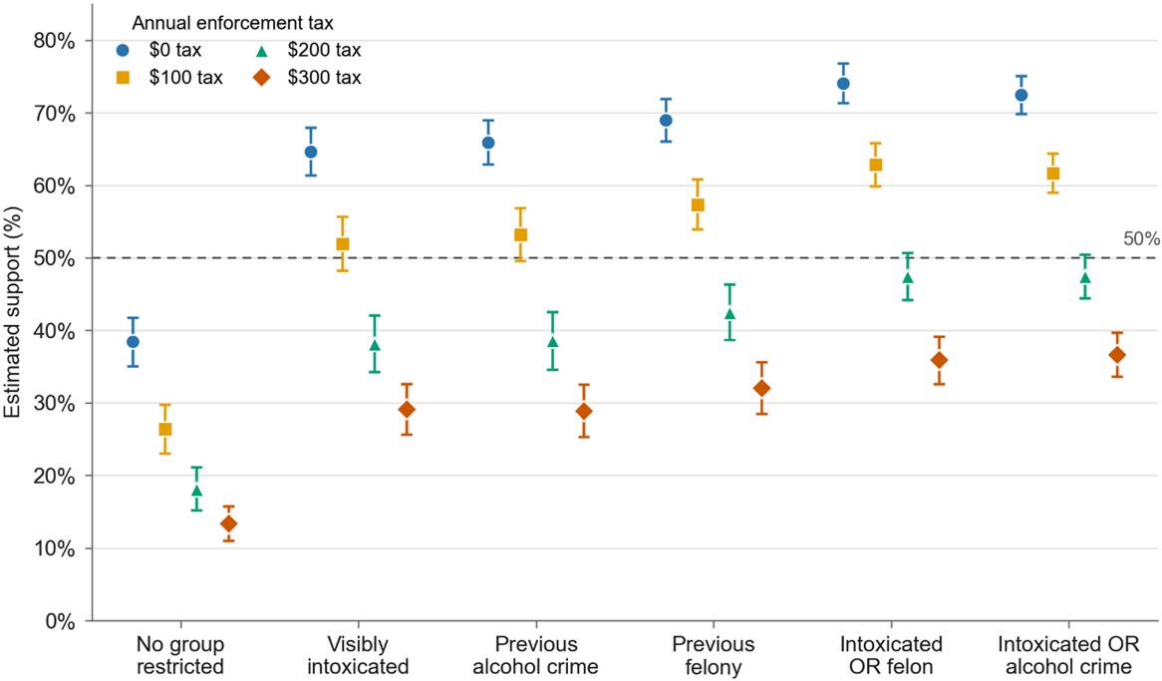
Weighted estimates of the percent of US adults supporting firearm possession restrictions in large venues, by restricted group and annual individual tax for measure enforcement. Notes: Points are weighted mean predicted support for a single-venue measure applying to large venues (with no restrictions in the other 2 venue types), whiskers are standard errors. Dashed line marks majority (50%) support.

Importance weights capture the relative influence of each attribute on respondents’ choices: for each respondent, the range of partworth utilities within an attribute expressed as a percentage of the summed ranges across all attributes (Appendix Equation 1). Importance weights are distinct from the predicted support probabilities reported in Figures 1 and 2 and Appendix Table S2.

Figure 1 shows the estimated percentage of the voting-age population that supports a tax-free measure to bar a certain group of people from possessing firearms in public places, by type of group and venue size. There is majority public support (56%-74%, depending on the restricted group and venue size) for the regulation of firearms where alcohol is consumed. This finding is largely consistent with existing opinion polls.^8^

Figure 1 reveals a strong positive correlation between venue size and support. For example, barring felons from possessing firearms rises from 57% in small to 63% in medium to 69% in large venues. Holding the restricted group constant, the increase in support from small to large venues is statistically significant at the 5% level for 3 of the 5 groups (felons, alcohol criminals, and either intoxicated OR felons); the increases are 12.0 (*P* = 0.007), 10.0 (*P* = 0.023), and 12.6 (*P* = 0.003) percentage points, respectively. The figure also shows that public support is strongest (74.1%) for a restriction of either intoxicated people or felons in large venues.

While Figure 1 confirms the popularity of restricting at least some people from carrying firearms in public places suggested by opinion polls and provides a useful but unsurprising moderator of such popularity in venue size, the main contribution of this paper is in Figure 2, which documents a strong negative effect of increasing enforcement costs on popular support for the restrictions we study. Figure 2 focuses on large venues for clarity (analogous effects occur for the other 2 venue sizes), and shows the estimated percentage of the voting-age population that would support restricting different types of people from carrying firearms there when such a restriction involved a cost of enforcement in the form of an additional annual tax. The $0-tax estimates are identical to the large-venue estimates in Figure 1 and show robust support for measures that are free to enforce. But as tax increases required to support enforcement increase from $0-$300, support falls precipitously for all measures. Holding the restricted groups constant, differences in support between adjacent tax levels are statistically significant in 13 of the 15 comparisons. (See Appendix Table A4).

While “free” restrictions in large venues are broadly popular with estimated popular support of over 65%, highly statistically significant regardless of the restricted group, Figure 2 shows that only 3 “costly” measures have statistically significant support above the majority of the population: restrictions on felons only (57.4% support, SE 2.5%), restricting felons and intoxicated people (62.9% support, SE 2.9%), and restricting both those with prior alcohol-related crimes and intoxicated people (61.7% support, SE 2.7%).

Another way to quantify the effect of increasing enforcement costs on the popular support for the restrictions we study is to calculate the “willingness to pay” or “WTP”, ie, the monetary equivalent of the value respondents place on each potential restriction. Appendix Table S3 reports the corresponding median marginal WTP for each restriction: median valuations range from $43- $72 per year of additional tax in small and medium venues and from $76-$109 in large venues, rising with venue size.

Table 2 shows results of the individual-level regression models of each respondent's posterior-mean predicted support for each potential restriction in large venues, fixing the tax at $100. The results of the same regressions for small and medium venues are similar and available in Table A4 in the Appendix. Because the regressions treat each respondent's posterior-mean predicted support as data, uncertainty from the choice model is not propagated into their standard errors. The “None” column indicates support for $100 tax alone.

**Table 2.** Regression results of individual support for different restrictions in large venues, given a $100 tax.

	Group of people restricted by the measure											
	None (tax increase only)		Currently intoxicated		Prior alcohol conviction		Felon		Intoxicated or felon		Intoxicated or alcohol conviction	
Individual Characteristics	coeff	*P*-value	coeff	*P*-value	Coeff	*P*-value	coeff	*P*-value	coeff	*P*-value	coeff	*P*-value
Intercept	21.0	0.001	38.9	0.000	36.9	0.000	47.2	0.000	43.2	0.000	47.5	0.000
Age 30 to 44	−10.1	0.001	−6.3	0.083	−8.3	0.017	−6.0	0.097	−4.4	0.215	−5.3	0.177
Age 45 to 59	−27.4	0.000	−30.7	0.000	−32.4	0.000	−31.4	0.000	−27.3	0.000	−29.8	0.000
Age 60 and over	−34.9	0.000	−41.4	0.000	−45.2	0.000	−46.4	0.000	−40.1	0.000	−38.1	0.000
Democrat female	11.2	0.015	29.3	0.000	36.4	0.000	28.5	0.000	37.7	0.000	25.6	0.000
Democrat male	−1.1	0.810	15.1	0.005	21.6	0.000	14.4	0.007	25.4	0.000	17.0	0.004
Independent female	0.1	0.984	6.6	0.320	14.8	0.021	5.3	0.419	16.0	0.016	5.3	0.464
Independent male	−10.1	0.033	−5.7	0.316	1.7	0.756	−6.3	0.259	2.6	0.639	0.1	0.987
Republican female	21.1	0.000	25.5	0.000	26.7	0.000	26.0	0.000	30.0	0.000	19.7	0.005
Gun owner	2.6	0.362	1.5	0.663	−0.5	0.873	−0.3	0.918	0.6	0.864	1.6	0.669
State gun grade	3.2	0.308	1.0	0.794	1.6	0.664	2.3	0.544	0.4	0.907	0.2	0.953
Log state pop density	16.1	0.023	3.2	0.707	4.4	0.590	−0.6	0.946	−0.6	0.943	1.8	0.844
Worry/gun violence	5.2	0.240	15.0	0.006	13.1	0.011	14.3	0.008	15.0	0.005	17.1	0.004

“None” means no restrictions in that venue size, and hence indicates support for $100 tax increase alone. Log state pop density has mean 7.2 and standard deviation of 1.8. Shooting worry is re-scaled to be between 0 and 1 (0 = ”Not Worried at All” to 1 = “Very worried”). Gifford grade is coded between 0 (“F”) to 6(“A”) such that higher grade has a better grade.

Holding all other characteristics constant, Table 2 shows that being younger, a Democrat, or female all increase support by double-digit percentage points. Regarding psychographics, only worrying about becoming a gun violence victim increases support for restrictions significantly in the statistical sense. Population density, state Giffords grade, and gun ownership have no significant effects on support. Looking more closely, 3 levels of support exist based on the combination of political affiliation and gender: (1) Republican men and Independents of both genders support restrictions the least; (2) Democrat men exhibit higher support, between 14% and 25% points (depending on the restricted group) higher than 1, and (3) Democrat and Republican women are the strongest supporters of restrictions, between 20% and 37% points more (again depending on the restricted group) than 1. All the correlation patterns discussed in this paragraph are similar in the small- and medium-venue regressions (see Table A4 in the Appendix).

## Discussion

Our study replicates the result of prior opinion polls that, as long as the restrictions are free to implement, there is widespread support for interventions that ban firearms in most locations where alcohol is served. This widespread support for restrictions is consistent with the results of the 2023 National Gun Policy Survey, which found that 49% of respondents favored banning firearms in restaurants, 75% in bars, and 71% in sports stadiums.^8^ Adding nuance to this baseline support of “free to implement” restrictions, we find that preferences for the restrictions increase with the size of the venue, but did not vary much with the type of person (felon, etc.) who would be subject to the law.

However, estimated popular support declines below majority once our respondents encounter an enforcement cost in the form of an annual nominal tax increase. Specifically, we find that support for restricting some people from possessing firearms in public places that serve alcohol is weaker than respondents’ dislike of a $100 tax increase. In other words, potential voters value most of the restrictions we study less than $100 annually, and would rather allow firearm possession by licensed individuals in public places that serve alcohol than pay that much more in taxes every year. The only restrictions that retain majority support even with an enforcement cost of additional $100 in taxes are restrictions on felons or broad restrictions (both felons and intoxicated people, or both intoxicated people or people with alcohol-related convictions) and only in large venues.

Regarding people's WTP for enforcement of the referendum, our results suggest that popular support is sensitive to the price potential voters may be asked to pay for enforcement in the form of annual taxes. Specifically, we find that as tax increases required to support enforcement increase from $0- $100, support for most restrictions falls precipitously, leaving most of the estimated support below the majority level. This finding contrasts with recent studies that used contingent valuation methods to assess Americans’ and California residents'WTP for preventing gun violence. These studies found that at the national level, people were willing to pay $744 each year for a 20% reduction in gun violence, with higher income, education, living in the northeast region and non-rural areas, more liberal political views (political party affiliation was not asked), and concern about becoming a victim of gun violence associated with higher WTP.^22^ In contrast, the WTP we calculate is mostly below $100 per year and occasionally (mostly for broad restrictions in large venues) just above $100.

A major difference between these studies and ours is that in the former, the choice respondents faced was between paying a tax and having population-level benefits (respondents were asked about support for preventing/reducing deaths stemming from firearm-involved homicides, suicides, mass shootings, or all 3 causes^29^). In our study, we only assessed support for a new law, which did not provide any indication of how it might affect population health.

Two caveats apply to interpreting the cost attribute. First, respondents were told only that the tax was associated with the measure's enforcement, which likely combines beliefs about enforcement costs with more general tax aversion, and our design cannot separate the 2. Second, the design presented costs without any information about potential offsetting savings to law enforcement, emergency medical services, or hospitals from fewer firearm-related incidents, or about population-health benefits; support might have been higher under such framings.

Some laws targeting the intersection of alcohol and firearms may have positive impacts on population health. For example, ERPO laws have been found to contribute to reductions in homicide and suicide^30^, with 1 study estimating 1 life was saved for every 10-to-12 gun removals in that state.^31^ Evaluations of these laws also show that a sizeable proportion of perpetrators were under the influence of alcohol or other drugs.^31^ The potential impact of removing firearms from those with a history of alcohol abuse was previously informed by an agent-based model exploring the (simulated) scenario where firearm purchases were restricted for individuals with previous drug or alcohol charges. Results suggest that such an intervention had the potential to reduce firearm homicide by as much as 1.0% and suicide by as much as 3.0%.^32^ Given the objective benefit of the restrictions, these results contribute to understanding of restrictions that are actually politically feasible because they enjoy majority support in the population.

Two of the scenarios studied also raise implementation questions. Federal law already generally prohibits firearm possession by people with felony convictions, so the felon scenario is best interpreted as a familiar benchmark against which support for novel, alcohol-specific restrictions can be compared, although venue-specific state measures could add enforcement mechanisms and penalties. Restrictions on visibly intoxicated people raise the question of who assesses intoxication and who bears responsibility for enforcement, challenges analogous to those long documented for “obviously intoxicated” standards in dram-shop liability and responsible beverage service laws. These implementation questions are one reason enforcement costs, and the public's willingness to bear them, are especially important for policy design.

Our study did not find statistically significant differences in support between gun owners and non-owners. This is consistent with studies showing that, controlling for political affiliation, gender, and other characteristics, gun ownership had no significant independent effect on support for restrictions in alcohol-serving venues. It also echoes findings by Hartley and Davila,^33^ who found that identity-based attitudes were stronger predictors of gun policy positions than ownership status alone.

Our results regarding the interaction of gender with political affiliation provide additional insights into support for gun violence protections. A well-documented gender gap exists in gun policy attitudes, with women consistently expressing higher support for gun restrictions than men across a wide range of studies. Crifasi et al.^34^ found that women supported 20 out of 21 gun policies at higher rates than men, with differences exceeding 10% points on several measures. Our finding that Republican women converge with Democratic women, rather than with Republican men, as the strongest supporters of restrictions aligns closely with work by Hansen and Dolan,^7^ who found that Republican women were approximately 12% points more supportive of gun control than Republican men, with a smaller gender gap among Democrats. They suggest Republican women experience competing pressures from gender identity (which pulls toward supporting gun restrictions) and partisan identity (which pulls away from it), with gender emerging as the dominant influence on this issue. This may also explain the somewhat surprising finding that political independents in our study did not fall between Democrats and Republicans, as is typically assumed. Research has shown that independents’ gun policy views tend to be more variable and context-dependent than those of strong partisans, whose positions remain relatively stable regardless of specific policy framing.^35^ In the context of this study, attitudinal and identity-based factors, particularly favorability toward the NRA, may have been important unmeasured variables.^33^

Finally, we find a strong negative effect of age on the support for the restrictions we study, but this finding is preliminary because our internet-based respondent sampling under-samples older people, so the correlation we find is based on a very small sample.

### Limitations

This study has several limitations. While DCEs have been used to predict behaviors such as purchasing expensive household items or choosing among health insurance features, there is no way to assess how the individuals surveyed here would actually vote in a real referendum. The sample size was relatively small, so some differences (such as those between different race/ethnic groups) likely had insufficient power to differentiate among participants. Finally, participants were not randomly selected from the US population, so weighted averaging was necessary to strengthen external validity of population and sub-population level predictions. Despite attention checks and other procedures that the survey provider includes to assure high-quality responses, there is no way to ascertain how selection bias (among others) may have affected results. In addition, because the individual-level regressions treat each respondent's estimated support as data, uncertainty from the choice model is not carried into those standard errors, and subgroup analyses should therefore be considered exploratory. Population-level estimates should be read as weighted sample estimates rather than precise population parameters.

## Conclusions

This study finds that there is substantial support for free-to-implement regulations that limit the carrying of firearms in places where alcohol is served, and this support increases with venue size. However, support drops substantially once we introduce an enforcement cost in the form of an annual nominal tax increase of only $100. Explaining and framing implementation costs is thus in itself an important consideration for lawmakers considering the formulation of new policies to reduce alcohol and firearm-related injuries and deaths. The finding that gender moderates the effect of political affiliation on support suggests that targeted messaging strategies may be more effective than broad partisan appeals in building coalitions for legislation on the intersection of alcohol and firearms. Partnerships with responsible gun owner groups could be helpful in translating these findings into practice.

## Supplementary Material

qxag200_Supplementary_Data
